# The Effect of Maturity Stage on Polyphenolic Composition, Antioxidant and Anti-Tyrosinase Activities of *Ficus rubiginosa* Desf. ex Vent. Extracts

**DOI:** 10.3390/antiox13091129

**Published:** 2024-09-18

**Authors:** Ghaid W. A. Abualzulof, Samir Scandar, Ina Varfaj, Vanessa Dalla Costa, Roccaldo Sardella, Raffaella Filippini, Anna Piovan, Maria Carla Marcotullio

**Affiliations:** 1 Department of Pharmaceutical Sciences, University of Perugia, Via Fabretti 48, 06123 Perugia, Italy; ghaidwa.abualzulof@dottorandi.unipg.it (G.W.A.A.); samir.scandar@dottorandi.unipg.it (S.S.); ina.varfaj@dottorandi.unipg.it (I.V.); roccaldo.sardella@unipg.it (R.S.); 2 Department of Pharmaceutical Sciences, University of Padua, Via Marzolo, 5, 35131 Padua, Italy; vanessa.dallacosta@phd.unipd.it (V.D.C.); raffaella.filippini@unipd.it (R.F.)

**Keywords:** *Ficus rubiginosa*, polyphenolic compounds, antioxidant activity, DPPH, FRAP, ABTS, tyrosinase

## Abstract

*Ficus* spp. are often used as food and in traditional medicine, and their biological activities as anti-inflammatory and diuretic, for wound healing, and as antimicrobial agents have been largely reviewed. The aim of this work was to investigate the polyphenol content and the antioxidant and anti-tyrosinase properties of the extracts from *F. rubiginosa*, a very poorly explored *Ficus* species. For this purpose, *F. rubiginosa* leaves were collected at three different maturity stages (H1, H2, and H3), and the environmentally sustainable methanolic extracts were evaluated for the total phenolic content (TPC), total flavonoid content (TFC), and total catechins content (TCC). The polyphenolic profile was studied using HPLC-UV/DAD and UHPLC-MS, and the antioxidant activity was determined in vitro using DPPH, FRAP, and ABTS assays. The study showed that the H2 extract had higher TPC and TFC values (113.50 mg GA/g and 43.27 mg QE/g, respectively) and significant antioxidant activity. Therefore, the H2 extract was selected to study the anti-tyrosinase activity. The results also showed that H2 was able to bind and inhibit tyrosinase, with rutin being the compound responsible for the measured activity on the enzyme.

## 1. Introduction

*Ficus* (Moraceae) is a large genus comprising more than 800 species [1] that are either woody, broad-leaved or evergreen trees, shrubs, or herbs. Components of this family are primarily found in tropical and subtropical regions [2]. Many *Ficus* spp. have been and are still used as food or medicinal plants. For example, *F. religiosa* [3], a tree that is considered sacred by both Buddhists and Hindus [4], is used in Ayurveda to treat diabetes and urinary disorders [5], whereas *F. hispida* is used traditionally in China and India as a remedy for skin disorders and respiratory and urinary diseases [6]. *F. carica* is perhaps the most famous species. It is widely cultivated for its fruits, commonly called ‘figs’, and is economically important. Furthermore, it has also been traditionally used as a laxative, expectorant, and diuretic [7].

*Ficus* species are also characterised by several bioactive compounds, such as terpenoids, furocoumarins, alkaloids, and polyphenols [8,9].

Despite the significant amount of information about *Ficus* spp., little has been reported about the phytochemistry of *F. rubiginosa* Desf. ex Vent. [10].

*F. rubiginosa*, also known as *Mastosuke rubiginosa* (Desf. ex Vent.) Raf. or *Urostigma rubiginosa* (Desf. ex Vent.) Dasf. [1], is commonly known as ‘Port Jackson fig’ or ‘rusty fig’ due to the colour of the leaves’ lower hairy surface. It is an evergreen tall tree native to Australia that begins its life as an epiphyte but later produces thick aerial roots that provide strength and allow it to grow faster. Its leaves are obovate, ovate, or elliptic, mostly 7–10 cm long and 5–6 cm wide; the upper surface is glabrous, while the lower surface is mostly hairy and rust-coloured. Its fruits (figs) are globose, 10–20 mm in diameter, yellow in colour that gradually turns to red, and usually verrucose [11].

Polyphenols are the largest constituents in *Ficus* spp. and are responsible for several of their biological activities. They represent an important bioactive class of secondary metabolites [12] that act as antiradicals and antioxidants [13] and can also be used for various purposes: for example, in skin care [14], to prevent metabolic syndrome [15], and to treat age-related diseases [16], to cite some. Kar et al. [17] studied the stimulating activity of *F. religiosa* polyphenols containing extracts on the thyroid, and others examined the antiproliferative activity of *F. glumosa* [18] and *F. awkeotsang* [19] and the antibacterial activity of *F. sycomorus* [20]. However, the antioxidant activity has been investigated the most [21,22,23,24,25,26]. It is well documented that the developmental stage influences the polyphenolic content [27,28,29]. Furthermore, Nadeem and Zeb evaluated the polyphenolic content in *F. carica* leaves at different stages of maturation [30].

It is well known that tyrosinases are an important class of enzymes responsible for melanin formation. In recent years, tyrosinase inhibitors have gained particular attention due to their use as depigmenting agents in treating skin disorders such as melasma, senile lentigos, and post-inflammatory hyperpigmentation [31]. Several tyrosinase inhibitors have been isolated from plants, and among them, polyphenols play an important role [32]. Moreover, among *Ficus* leaf extracts, *F. sur*, *F. carica*, and *F. sycomorus* leaf extracts have been found to exert tyrosinase inhibitory properties [33,34,35].

The present work aimed to study the polyphenol content and antioxidant activity of *F. rubiginosa* leaves methanolic extract at three different stages of maturity and to determine the polyphenol extract that contains the highest activity against tyrosinase enzyme.

## 2. Materials and Methods

### 2.1. Chemicals

Methanol (MeOH) and ethanol (EtOH) were purchased from VWR (Milan, Italy); MeOH and acetonitrile (ACN) for HPLC 99%, sodium nitrite (NaNO_2_), sodium hydroxide (NaOH), aluminium chloride (AlCl_3_, anhydrous sublimed), catechin (C), quercetin (Q), phosphate buffer (PBS), and potassium persulfate (K_2_S_2_O_8_) from Merck Life Science S.r.l. (Milan, Italy); formic acid (FA) and anhydrous sodium sulfate (Na_2_SO_4_) from CARLOERBA (Milan, Italy). Water for the HPLC analysis was purified using a Milli-Q water purification system from Millipore (Milan, Italy). LiChrosolv water, LiChropur formic acid, and LiChropur ammonium formate (HCO_2_NH_4_) (all LC-MS grade) were bought from Supelco^®^ Analytical Products Merck Life Science S.r.l. (Milan, Italy). Folin–Ciocalteu (FC) reagent, 2,2′-azino-bis-(3-ethylbenzothiazoline-6-sulphonate) diammonium salt (ABTS), 2,4,6-tris(2-pyridyl)-s-triazine (TPTZ), 6-hydroxy-2,5,7,8-tetramethyl-2-carboxylic acid (Trolox), 2,2-diphenyl-1-picrylhydrazyl (DPPH), ferric chloride (FeCl_3_), gallic acid (GA), rutin, ascorbic acid, DOPA, and mushroom tyrosinase (5370 units/mg) were purchased from Sigma-Aldrich (Milan, Italy).

### 2.2. Instrumentation

UV/Vis spectrophotometric assays were performed using a Sunrise™ Absorbance microplate reader (TECAN, Männedorf, Switzerland). All the spectrophotometric experiments were performed using disposable optical Corning^®^ 96-well plates from Merck Life Science (Merck KGaA, Darmstadt, Germany) and a Sunrise microplate reader (Tecan Italia S.r.l., Milan, Italy). The HPLC-UV/DAD analyses were performed using an Agilent 1100 HPLC Series System equipped with a degasser, quaternary gradient pump, column thermostat, and UV-Vis detector (Agilent, Santa Clara, CA, USA). UHPLC-MS analyses were performed on an Agilent 1290 Infinity II model combined with the Agilent 6550 mass spectrometer (Agilent, Santa Clara, CA, USA). A Gemini 5 μm C6-Phenyl column (250 × 4.6 mm) from Phenomenex (Torrance, CA, USA) was used for all the chromatographic separations.

### 2.3. Plant Material

The leaves of *Ficus rubiginosa* Desf. ex Vent. were collected at the Botanical Garden of Padua in April (H1), July (H2), and September (H3) 2022 and immediately oven-dried at 45 °C (Air Concept, Froilabo, Collégien, France). A voucher specimen (H0061262) was deposited at the Department of Pharmaceutical Sciences, University of Padua. A domestic food processor, Girmi Mod. TR20 (Girmi, Omegna (VB), Italy), was used to pulverise the leaves that were sieved, and the <710 μm granulometric fraction was collected.

### 2.4. Plant Material Extraction

The powdered leaves (4 g) were macerated using MeOH, EtOH, aqueous 80%, 70%, and 60% EtOH (*v*/*v*) (3 × 40 mL) overnight, at room temperature (r.t.). All the organic extracts were dried over anhydrous Na_2_SO_4_, filtered, and evaporated under reduced pressure.

### 2.5. Evaluation of the Total Phenol Content (TPC), Total Flavonoid Content (TFC), and Total Antioxidant Capacity (TAC)

#### 2.5.1. Evaluation of Total Phenol Content (TPC)

Spectrophotometric assays were carried out following a procedure described in previous works [36,37] with only a few modifications. The Folin–Ciocalteau (FC) method was used to determine the TPC. Briefly, the three harvested extracts (H1, H2, and H3) were dissolved in plain MeOH to prepare a 1.0 mg/mL final concentration. Next, 100 μL of the extract and 750 μL of the 10-fold diluted FC reagent were mixed and kept for 10 min at r.t. The obtained solution was treated with 750 μL aqueous Na_2_CO_3_ (2% (*w*/*v*)) and left at r.t. for 3 h. The absorbance was read at 765 nm. The TPC values were calculated using a calibration curve prepared with gallic acid (GA) solutions (calibration curve in the range 0.01–0.25 mg/mL; R^2^ > 0.999) previously treated in the same way as the studied samples. For each extract, all the measurements were made with 0.2 mL solutions. The TPC results are expressed as follows: mg of gallic acid equivalents (GAE)/g dry extract.

#### 2.5.2. Evaluation of the Total Flavonoid Content (TFC)

The total flavonoid content (TFC) was estimated by applying the well-known AlCl_3_ method [38], with minor modifications. Briefly, a regression curve (R^2^ = 0.999) was built up using quercetin as a surrogate standard (in the 0.03–0.10 mg/mL concentration range). The stock solution of quercetin was prepared at 1.0 mg/mL concentration with 50% (*v*/*v*) aqueous MeOH. Next, 2 mL of MeOH were added to either 0.5 mL of the quercetin solution or the extract solutions (each extract, in the range 0.4–0.65 mg, was preliminarily diluted with 2.0 mL of 50% *v*/*v* aqueous MeOH). For the analysis, 0.2 mL of AlCl_3_ (anhydrous sublimed, 10% *w*/*v* in water) were added soon after the preparation of these solutions, which are referred to as the initial solutions. After 3 min, MeOH was added until a total volume of 5.0 mL was reached. These solutions were then vortexed for 10 s and kept in the dark for 40 min before analysis. The same procedure was followed for each solution used to build up the calibration curve, and the absorbance was read at 415 nm. The TFC results are expressed as follows: mg quercetin equivalents (QE)/g of dry extract. All the analyses were performed in triplicate.

#### 2.5.3. Evaluation of the Total Catechin Content (TCC)

The TCC was spectrophotometrically evaluated using the NaNO_2_/AlCl_3_ assay [39] with subtle modifications. A stock solution of catechin (5.0 mg/mL in 50% *v*/*v* aqueous MeOH) was prepared. A regression curve (R^2^ = 0.999) was created with catechin as a surrogate standard (in the 0.10–0.30 mg/mL concentration range) and used to measure the TCC of all catechin-type compounds. Next, 2 mL of MeOH were added to either 0.5 mL of the extract solutions or the standard solution (each extract, in the range of 0.4–0.65 mg, was preliminarily diluted with 2.0 mL of 50% aqueous MeOH). To this, 0.15 mL of aqueous NaNO_2_ (1.0 M) were added and sequentially vortexed for 10 s. After 3 min, 0.15 mL of AlCl_3_ (anhydrous sublimed, 10% *w*/*v* in water) were added, and the resulting mixture was vortexed for 10 s. Afterwards, 1.0 mL of aqueous NaOH (1.0 M) was added to the mixture. Then, MeOH was added until a total volume of 5.0 mL was reached; the obtained solution was vortexed for 10 s and kept in the dark for 40 min before analysis. The absorbance was read at 510 nm. The same procedure was applied to each solution used to build up the calibration curve. The results were expressed as mg of catechin equivalents (CE)/g of dry extract. The procedures were carried out in triplicate.

#### 2.5.4. Evaluation of the Total Antioxidant Capacity (TAC) Using the Frap Method

For the preparation of the FRAP reagent, the following solutions were mixed: 2.5 mL of a TPTZ solution (10 mM) in HCl (40 mM, aqueous) and 2.5 mL of an FeCl_3_ aqueous solution (20 mM), mixed with 25 mL of aqueous NaOAc (300 mM, pH 3.6), following a procedure previously reported [36,37] and modified. H1, H2, and H3 were dissolved in plain MeOH in order to obtain a 1.0 mg/mL final concentration. The assay was carried out by mixing 100 μL of diluted methanolic extract, 100 μL of distilled water, and 1.5 mL of FRAP reagent and maintaining the solution at r.t. in the dark for 4 min. The absorbance was read at 593 nm. The TAC values were determined from a calibration curve prepared with Trolox (as the surrogate standard) solutions, previously treated using the same procedure as that used for the studied sample (calibration curve in the range 0.0125–0.25 mg/mL; R^2^ > 0.999). For the measurements, 0.2 mL of the solutions were used. All the experiments were performed in triplicate for each extract, and the results are expressed as follows: µmol of Trolox equivalents (TE)/g dry extract.

#### 2.5.5. Evaluation of the Radical Scavenging Capacity (RSC) by the DPPH Method

The RSC was measured using the DPPH method, slightly modifying a previously described procedure [36,37]. DPPH was solubilised in HPLC-grade EtOH until a concentration with an absorbance of 0.65 (±0.02) at 517 nm was reached, and the absorbance value was stabilised within 2 h. Each extract was dissolved in plain MeOH in order to obtain a 1.0 mg/mL final concentration. Next, 50 µL of the methanolic extract were added to 2.95 mL of the stabilised DPPH solution and maintained at r.t. in the dark for 30 min. The absorbance was read at 517 nm. The RSC was determined from a calibration curve prepared using Trolox (surrogate standard) solutions, previously treated by applying the same procedure as that used for the studied sample (calibration curve in the range 0.025–0.25 mg/mL; R^2^ = 0.998). For the measurements, 0.2 mL of the solutions were used. All the experiments were performed in triplicate for each extract, and the results were expressed as follows: µmol of Trolox equivalents (TE)/g dry extract.

#### 2.5.6. Evaluation of the Radical Scavenging Capacity (RSC) Using the ABTS Method

The ABTS method was used as a complementary method to DPPH to determine the RSC. This assay was evaluated as described in [36,37] with few modifications. Two volumes of an aqueous solution of ABTS^+^ (0.36% (*w*/*v*)) and one volume of a 0.2% aqueous solution of K_2_S_2_O_8_ were mixed. The flask was covered with aluminium foil and left overnight at r.t. in the dark. The obtained ABTS^•+^ solution was diluted with EtOH until the absorbance was 0.70 (±0.05) at 734 nm. Each extract was then solubilised, as reported in Section 2.5.1. To the methanolic extract (0.06 mL), 4.0 mL of the ABTS^+^/EtOH solution was added, and the mixture was left standing in the dark for 6 min. The absorbance was read at 734 nm. The radical scavenging capacity was extrapolated from a calibration curve prepared with Trolox (surrogate standard) solutions, previously treated using the same procedure as the studied sample (calibration curve in the range 0.05–0.50 mg/mL; R^2^ > 0.997). For the measurements, 0.2 mL of each solution were used. All the experiments were performed in triplicate for each extract, and the results are expressed as follows: µmol of Trolox equivalents (TE)/g dry extract.

### 2.6. HPLC-UV/DAD and UHPLC-MS Analysis of the Extracts and for the Tyrosinase Binding Tests

For the HPLC-UV/DAD analyses, the column was employed at 40 °C. The optimised gradient program was as follows: eluent A water with 0.15% (*v*/*v*) acetic acid, eluent B acetonitrile; 97% A at 0–6 min, 75% A at 15 min, 75% A at 20 min, 20% A at 30 min, and 97% A at 40 min. The flow rate was 1 mL/min, with an injection volume of 10 μL; chromatograms were acquired at 265 and 365 nm; and UV–Vis spectra were recorded in the 190–700 nm range.

Rutin was quantified by acquiring the chromatograms at 365 nm and using a 1 mg/mL standard solution in MeOH. The calibration curve (R^2^ = 0.999) was obtained in the 2–50 μg/mL concentration range, with five concentration levels. The analysis was performed in triplicate, and the results were expressed as mean ± standard deviation (SD).

The optimised gradient program for UHPLC-MS analyses was as follows: eluent A water with 0.1% (*v*/*v*) formic acid, eluent B acetonitrile; 0–8 min, 97% A; 8–26.5 min, 75% A; 26.5–40 min, 20% A; 40–42 min 97% A. The flow rate was 0.75 mL/min, with an injection volume of 5 μL and the column temperature was kept at 40 °C. Chromatograms were acquired at 265 and 365 nm, and the UV–Vis spectra recorded in the 190–700 nm range. For MS detection, the Dual AJS ESI source operated in the negative ion mode. The parameters used were as follows: gas temperature: 300 °C; flow: 5 L/min, sheath gas temperature: 250 °C with a flow of 11 L/min. The nebuliser pressure was set at 35 psi, and the Capillary and Fragmentor voltages were 3500 V and 260 V, respectively. The MassHunter Workstation Data Acquisition 10.0 (Agilent Technologies Inc., Santa Clara, CA, USA) program was used for data acquisition, while the MassHunter Qualitative Analysis 10.0 (Agilent Technologies Inc., Santa Clara, CA, USA) software was used for data processing. 

### 2.7. Evaluation of the Tyrosinase Affinity Using the Target Binding^®^ Technology 

The *F. rubiginosa* constituents’ affinity to tyrosinase was studied using the Target Binding^®^ technology [40], a method commonly used to pre-select the inhibitor candidates in complex extracts, with few modifications. Mushroom tyrosinase was prepared at 2000 U/mL in 50 mM phosphate buffer pH 6.8 (PBS). The tyrosinase solution was mixed with H2 extract and incubated at r.t. for 10 min. After this time, the mixture was filtered using a 5 kDa cut-off centrifugal filter. The unbound compounds were eliminated (see Section 3.4 for details) through several washings, and the target–ligand complexes were solubilised in water. By adding acetonitrile, the bounded compounds (see Section 3.4 for details) were recovered. The experiment was performed in triplicate. The ligands and raw extracts were analysed using HPLC-DAD.

### 2.8. Evaluation of the Anti-Tyrosinase Activity

The anti-tyrosinase inhibitory activity was spectrophotometrically investigated following a method previously described [41], with few modifications. l-DOPA was used as a substrate for the tyrosinase. The assay mixtures, freshly prepared, consisted of 300 µL of test solution in 0.05 M phosphate buffer pH 6.8 (PBS) and 10 µL of enzyme solution (2000 U/mL in PBS). After preincubation at r.t. for 10 min, a 300 µL substrate solution (0.1 M l-DOPA in PBS) was added to start the reaction. This mixture was incubated at r.t. for 20 min, and the absorbance at 465 nm was measured with a Shimadzu UV-1800 spectrophotometer (Shimadzu Italia, Milan, Italy). Ascorbic acid, a known tyrosinase inhibitor, was used as a positive control. The tyrosinase inhibitory activity was expressed as the percentage inhibition of tyrosinase enzyme in the assay system used and calculated as follows:

$$\%\;\mathrm{antityrosinase}\;\mathrm{activity}=100\;\frac{\left(A-B\right)-\left(C-D\right)}{\left(A-B\right)}$$

where: A is the UV absorbance of a mixture containing water, tyrosinase, and substrate; B is the UV absorbance of a mixture containing water and PBS; C is the UV absorbance of a mixture containing extract, tyrosinase, and substrate; and D is the UV absorbance of a mixture containing extract and PBS.

Various concentrations of the samples were studied to obtain the tyrosinase inhibitory activity percentage. The IC_50_ value, which is the concentration required for a 50% tyrosinase inhibition, was determined using the dose–response curves of each sample via the GraphPad Prism (version 10.1.2, GraphPad Software, San Diego, CA, USA). Three independent experiments were performed in duplicate.

### 2.9. Statistics

All the analyses were performed in triplicate, and the values were reported as the mean ± standard deviation (SD) of the three independent analyses. The Statistica 12.0 software (StatSoft GmbH, Hamburg, Germany) was used to perform the statistical analyses. The significant differences among the studied parameters were analysed using a one-way ANOVA, followed by a post hoc Tukey’s Honestly Significant Difference (HSD) test at a significance level of *p* ˂ 0.05. The correlation between the total polyphenol content, DPPH antioxidant capacity, and FRAP-reducing power was evaluated through a Pearson correlation test.

## 3. Results and Discussion

Polyphenols are the most widespread secondary metabolites in flowering plants, where they are involved in the chemical defence against predators [42]. They are potent antioxidant compounds that can prevent, inter alia, the development of severe disorders, including cancer and cardiovascular-related diseases, in humans [43]. In plants, they are important modulators of cell signalling pathways [44]. Among polyphenols, flavonoids are, perhaps, the class with the most documented compounds with nutraceutical importance [45,46,47].

Phenolic composition results from genetic and environmental factors [48,49], such as plant genotypes, growth stages, seasons, and eco-geographical conditions. Therefore, identifying and quantifying the major phenolic compounds in a plant forms the basis for the rationalisation of its biological effect, often derived from the compounds’ antiradical, antioxidant, and anti-inflammatory activities.

*Ficus* spp. have been reported as a good source of phenolic compounds, such as coumarins and flavonoids [8].

Due to the lack of information on *F. rubiginosa* phytochemistry, we undertook this study to measure the phenolic content, antioxidant capacities, and anti-tyrosinase enzyme activities of the extracts from *F. rubiginosa* leaves collected at three different maturity stages: spring (H1), summer (H2), and autumn (H3).

### 3.1. Solvent Optimisation

Polyphenols are usually extracted from plants using methanol, ethanol, water, acetone, and their mixtures [50,51]. Methanol is often the best-extracting solvent for polyphenols and flavonoids [51,52], but it has been demonstrated that different plant materials can be better extracted using different solvents [53]. Given the recognition by the USA Food and Drug Administration (FDA) of ethanol as a ‘generally recognised as safe’ (GRAS) solvent, we carried out preliminary experiments to comparatively evaluate MeOH and EtOH, with and without mixture or not with water, to identify the best-extracting solvent in terms of polyphenol extraction yield. This preliminary screening of solvents was carried out on sample H1.

The extractions were performed by maceration to avoid any possible decomposition of metabolites. Table 1 shows the preliminary results obtained from the analysis of the prepared extracts.

The results indicate a statistically significant difference (*p* < 0.05) in the extraction yield obtained using MeOH and EtOH, while the extraction performed using EtOH-80, EtOH-70, and EtOH-60 showed a statistically significant difference only when compared to MeOH and EtOH. Meanwhile, the differences in TPC were statistically different only between MeOH and the other solvents. Moreover, the highest extraction yield was observed using EtOH-80, EtOH-70, and EtOH-60, and the highest total phenolic content (TPC) was obtained using methanol (*p* < 0.05) (Table 1). The difference between the yields and the TPC can be ascribed to the extraction of different classes of compounds in relation to polyphenols using aqueous ethanol [54]. Following these results, we choose methanol as the extracting solvent to evaluate the changes in polyphenol and flavonoid amounts during maturity.

### 3.2. Changes in TPC, TFC, and TCC over Time

Table 2 shows the percentage of the extraction yield obtained using MeOH for the three harvests. The results show that the maturity stage produced yields with a non-significant difference between H1 and H2 (*p* > 0.05). In contrast, a significant difference in the extraction yield was observed in H3 (*p* < 0.05).

The TPC, TFC, and TCC were determined on each of the three extracts. The TPC of the solubilised portion was evaluated using the FC method, with gallic acid (GA) as the standard. The results (Table 2) show that the time of the harvest significantly influences (*p* < 0.05) the TPC (expressed as mg GAE/g extract) value, which increased from spring (H1) to summer (H2) and decreased in autumn (H3). Various authors have also observed a TPC decrease in mature leaves. For example, Nadeem and Zeb found that the polyphenolic content in *F. carica* leaves decreased in 60-day old leaves [30]. Similarly, Liu et al. reported that the soluble TPC content decreases in old *Camellia sinensis* leaves following the normal increase of cell wall-bounded polyphenolics (lignins and condensed tannins) [55]. Chang et al. observed the same results in *Clausenia lansium* leaves [56]. This change can be ascribed to the fact that these two types of polyphenols share the same biosynthetic pathway, and during the maturation stages, precursors can change the flow from soluble to bounded phenols [57].

The TFC of all quercetin-type compounds was determined using the aluminium chloride (AlCl_3_) method, based on the formation of stable yellow complexes between AlCl_3_ and the C-4 keto group and either the C-3 or C-5 hydroxyl groups of flavones and flavonols [58]. Adding aluminium chloride to flavonoids leads to the formation of a new chromophore, specifically in quercetin- and rutin-like compounds. However, for catechin-type species, the absence of a carbonyl group stops the generation of the new chromophore. On the other hand, the addition of sodium nitrite in the assay protocol serves as a nitrating agent, and it is selective for aromatic vicinal diols, ultimately producing a flavonoid-nitroxyl derivative characterised by the appearance of additional absorption bands after about 320 nm, including one at about 510 nm. The appearance of these new red-shifted bands produces coloured nitrophenols [39], and catechin-type flavonoids can also be quantified in this way. These results show that TFC behaves like general polyphenols. In contrast, TCC tends to increase during this time, being significantly higher in H3 (*p* < 0.05) than in the other two extracts. Interestingly, our findings differ from those reported by other authors. For instance, Chang and Liu observed an increase of flavonoids (determined with a different analytical method) in mature leaves [55,56]. On the contrary, Anwar’s findings, obtained using the same methodology, align with our outcomes [59]. Interestingly, the TCC content does not follow the same pattern, as it increases during the three stages from 89.60 ± 0.41 (H1) to 101.67 ± 0.47 (H3). As per our knowledge, this is an unusual trend, as different authors have reported that catechin (flavanol) content usually follows the same trend as flavonoids and phenolics [55,60].

### 3.3. Evaluation of the Antioxidant Activity Using the DPPH, FRAP, and ABTS Methods

It is well known that different ‘chemical families’ of antioxidants can respond differently to different types of assays [61]. Therefore, the more formally correct way to evaluate a plant extract’s TAC (Total Antioxidant Capacity) is to perform assays based on different reaction mechanisms and consider cumulatively the results obtained from each of these. In this scenario, the use of spectrophotometric assays can allow the acquisition of important information easily and quickly.

Therefore, three different spectrophotometric assays, FRAP, DPPH, and ABTS, were performed to determine the TAC of the three extracts. The FRAP method is exclusively based on a single electron transfer (SET) mechanism, while the radicals characterising the DPPH and ABTS assays are recognised to activate mixed-mode hydrogen atom transfer (HAT) and SET reactions, depending on the applied experimental conditions [62,63].

Table 3 reports the results of the antioxidant activity determined on the three harvests using different methods.

The results show that the leaves collected in spring (H2) exhibited the highest antiradical and antioxidant activities in DPPH and FRAP assays, which was significantly different from H1 and H3 (*p* < 0.05).

Conversely, the youngest leaves (H1) demonstrated the lowest antioxidant activity in the DPPH and ABTS assays, which was also significantly different from H2 and H3 (*p* < 0.05).

**Figure 1:**
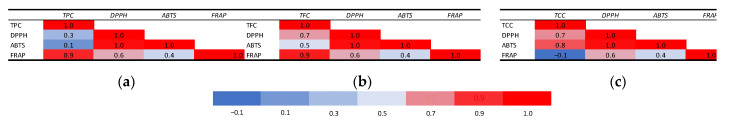
Pearson’s correlation coefficient between TPC (**a**), TFC (**b**), and TCC (**c**) vs. antioxidant activities.

The correlation between the antiradical/antioxidant activity and the different classes of phenols is reported in Figure 1.

The correlation shows that TPC and TFC have a very strong positive correlation with FRAP and a weak correlation with DPPH and ABTS assays. On the contrary, TCC shows a strong correlation with DPPH and ABTS and a very weak negative correlation with the FRAP assay. These data suggest that TFC and TCC are primarily responsible for the antioxidant of *F. rubiginosa* extracts.

### 3.4. Chromatography- and Mass Spectrometry-Based Analyses and Tyrosinase Binding Assay

Given that it has the highest TPC and TFC content and the best antioxidant activity, H2 was selected for the tyrosinase binding activity analysis.

A preliminary HPLC-DAD analysis was performed to obtain the chromatographic profile and define marker compounds for the tyrosinase affinity and anti-tyrosinase activity studies. The MS data acquired during the analysis of H2 revealed the presence of 21 compounds (Table 4). The species were identified using literature data [64,65] and the MassHunter Qualitative Analysis 10.0 software suite (Agilent, Santa Clara, CA, USA). The UHPLC-MS analysis was performed in the negative ionisation mode, as reported in Section 2.6.

The identified polyphenols belong to four different classes of compounds, i.e., hydroxybenzoic acids (e.g., hydroxybenzoic acid, gallic acid, protocatechuic acid, syringic, vanillic acid), hydroxycinnamic acids (e.g., 5-caffeoylquinic acid, ferulic acid, *p*-coumaric acid), flavonols (e.g., rutin, kaempferol, quercetin and quercetin derivatives), and flavones (e.g., apigenin). Furthermore, we identified morin, a characteristic flavonol of the Moraceae family. Finally, rutin and protocatechuic acid were found to be the most abundant compounds.

The potential of the constituents from the H2 extract against tyrosinase was investigated using the Target Binding^®^ approach, which allows the identification of the compounds that interact with an enzyme. This assay is based on the interactions of a given protein target with a plant extract. Additionally, ligand molecules were revealed through the HPLC-UV analysis: the comparison of the chromatograms representing the raw extract and the Target Binding^®^ samples shows the molecules bound or not bound to the enzyme during the incubation step.

**Figure 2:**
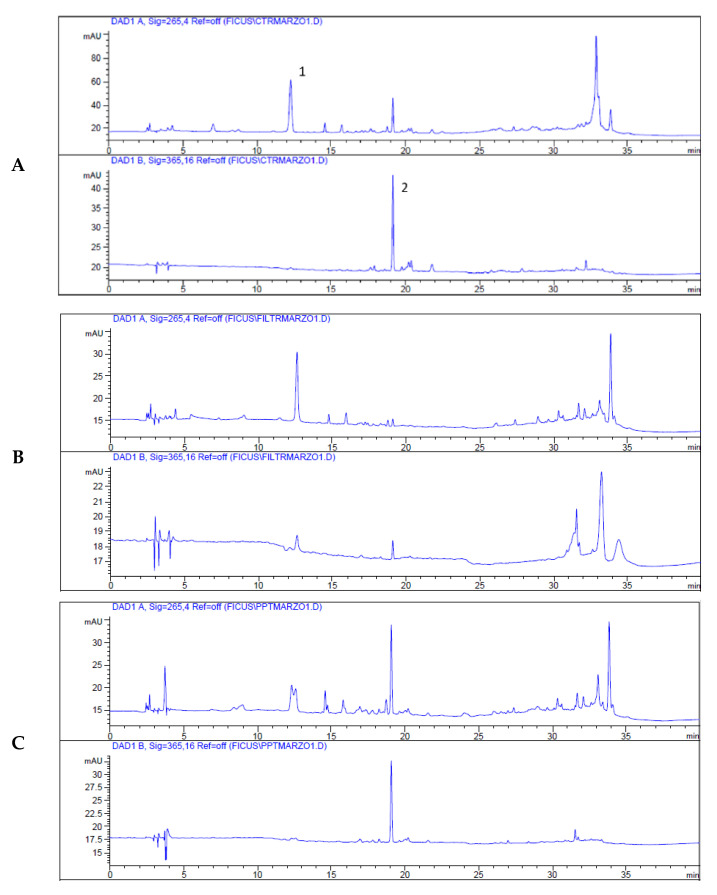
HPLC-UV chromatograms recorded at 265 (top) and 365 nm (bottom) of *F. rubiginosa* H2 extract (**A**), Target Binding^®^ filtrate (**B**), and Target Binding^®^ supernatant (**C**). The peaks are 1: protocatechuic acid and 2: rutin.

Figure 2 shows the comparison of the chromatograms of the H2 extract (A) and the filtrate containing the compounds not retained (B) and the supernatant containing the compounds retained by tyrosinase (C). The data suggest that rutin is the main or only compound in H2 that binds to the enzyme, whereas protocatechuic acid and chlorophylls do not.

**Figure 3:**
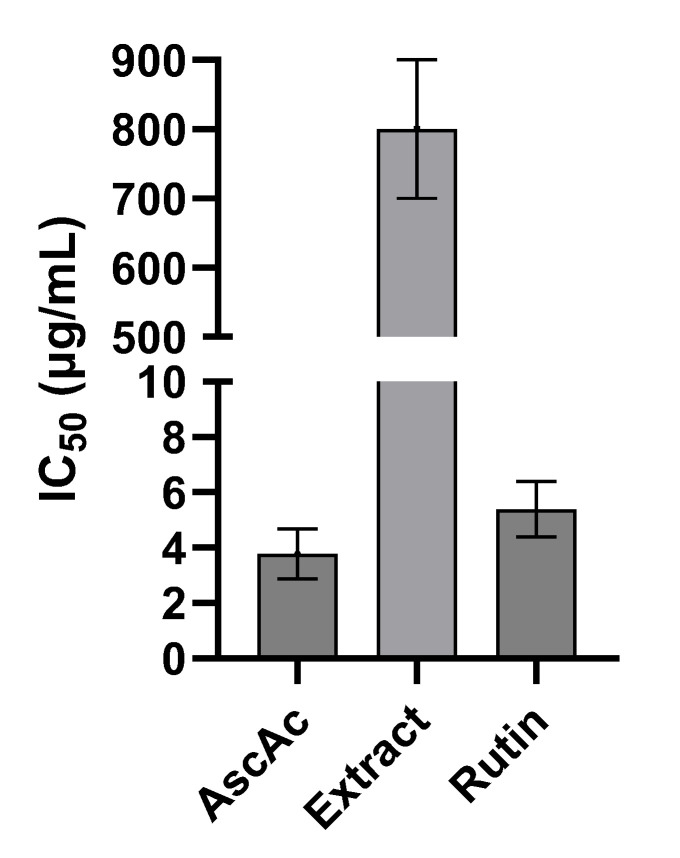
IC_50_ against tyrosinase of ascorbic acid (AscAc), *F. rubiginosa* H2 extract (Extract), and rutin standard.

Since rutin is a well-known tyrosinase inhibitor, it may be responsible for inhibitory effects on tyrosinase of *F. rubiginosa* H2 extract [66,67]. Therefore, the rutin content in the extract has been quantified, and the half-maximal tyrosinase inhibitory concentration (IC_50_) of the H2 extract and rutin standard have been assessed. The anti-tyrosinase activity of H2 extract was tested in the range of 100–1000 µg/mL, that is 0.7–7.2 µg/mL of rutin. The IC_50_ was determined as 800 µg/mL for the extract and 5.38 µg/mL for the rutin standard (Figure 3). Interestingly, the amount of rutin in 800 µg of H2 extract is 5.8 µg, comparable to the IC_50_ of standard rutin.

The results show that *F. rubiginosa* extract suppressed the tyrosinase activity, with rutin being responsible for it.

Although protocatechuic acid had no direct inhibitory effect on tyrosinase activity, it was reported to exert a potent anti-melanogenic effect by inhibiting the expression of melanogenic genes [68]. However, the presence of both rutin and protocatechuic acid in the extract adds value. *F. rubiginosa* may be an effective agent for hyperpigmentation disorders as it works either on tyrosinase activity or the melanogenic pathway.

## 4. Conclusions

*F. rubiginosa* is a scarcely studied species that must be examined in depth, as it has already been reported that its extracts exhibit an interesting antiviral activity [10]. The current study, as far as we know, appears to be the first phytochemical study of this species that examined the polyphenolic content of the leaf extracts. Although the present study has some minor concerns due to the fact that we took into consideration only single seasonality, we demonstrated that the maturity stage greatly influences the polyphenolic content, with the extract obtained from the July harvest (H2) exhibiting the highest TPC and TFC values. Furthermore, H2 also showed the highest DPPH, FRAP, and ABTS values, showing the extract’s potentially highest antioxidant capacity. This observation holds practical significance as it highlights the occurrence of a season-related type of phytocomplex. It also allowed us to identify the best harvesting time to obtain extracts with healthy properties. Accordingly, H2 was studied for its tyrosinase inhibition. The results showed that rutin plays a prominent role in the observed anti-tyrosinase activity and that the methanolic extracts of *F. rubiginosa* could be profitably used in the treatment of various skin diseases related to tyrosinase activity. As already stated, polyphenol content and profile are influenced by genetic and environmental factors. Therefore, we think it would be of great significance to enlarge the study, and it is our intention to further study polyphenol profile variation, taking into consideration different years and using leaf samples collected from plants grown in different habitats.

## Figures and Tables

**Table 1:** Yield and TPC obtained using different solvents on H1 leaves. Results, reported as mean value ± standard deviation (*n* = 3), are expressed on extract dry weight.

	Yield%	TPC (mg GAE/g)
MeOH	6.41 ± 0.40 *^a^*	93.71 ± 5.15 *^a^*
EtOH	4.44 ± 0.42 *^b^*	31.08 ± 2.81 *^b^*
EtOH-80	9.06 ± 0.24 *^c^*	43.50 ± 3.04 *^c^*
EtOH-70	10.6 ± 0.28 *^c^*	40.39± 0.12 *^c^*
EtOH-60	10.6 ± 0.57 *^c^*	37.65± 3.77 *^b^*

Different superscripted letters in the same column indicate significant differences (*p* < 0.05).

**Table 2:** TPC, TFC, and TCC obtained using MeOH as the extraction solvent. Results, reported as mean value ± standard deviation (*n* = 3), are expressed on the extract dry weight.

Harvest	Yield%	TPC (mg GAE/g)	TFC (mg QE/g)	TCC (mg CE/g)
H1	6.41 ± 0.40 *^a^*	93.71 ± 5.15 *^a^*	35.67 ± 1.76 *^a^*	89.60 ± 0.41 *^a^*
H2	7.02 ± 0.33 *^a^*	113.50 ± 3.55 *^b^*	43.27 ± 0.23 *^b^*	95.39 ± 0.54 *^b^*
H3	5.26 ± 0.42 *^b^*	75.69 ± 0.96 *^c^*	35.26 ± 0.59 *^a^*	101.67 ± 0.47 *^c^*

Different superscripted letters in the same column indicate significant differences (*p* < 0.05).

**Table 3:** In vitro antioxidant activity measured with the DPPH, FRAP, and ABTS assays of the three methanolic harvested extracts. Results, reported as mean value ± standard deviation (*n* = 3), are expressed on dry weight.

Harvest	DPPH (mg TE/g)	FRAP (mg TE/g)	ABTS (mg TE/g)
H1	546.26 ± 11.64 *^a^*	1.96 ± 0.05 *^a^*	463.36 ± 7.82 *^a^*
H2	721.65 ± 16.22 *^b^*	2.64 ± 0.01 *^b^*	579.84 ± 6.84 *^b^*
H3	675.43 ± 9.81 *^c^*	1.87 ± 0.01 *^a^*	573.02 ± 6.22 *^b^*

Different superscripted letters in the same column indicate significant differences (*p* < 0.05).

**Table 4:** Polyphenols identified in H2 extract by UHPLC-MS analysis.

Peak n°	Rt min.	[M-H]^−^(*m/z*)	Formula	Expected Mass	Score	Error (In ppm)	Compound
1	4.08	193.0694	C_8_H_10_N_4_O_2_	194.0804	98.60	0.19	Caffeine
2	4.43	137.0256	C_7_H_6_O_3_	130.029	91.52	8.58	*p*-Hydroxybenzoic acid
3	6.41	191.0212	C_6_H_8_O_7_	192.0825	88.92	7.95	Citric Acid
4	9.55	169.0157	C_7_H_6_O_5_	170.0215	89.36	8.49	Gallic Acid
5	16.42	153.0208	C_7_H_6_O_4_	154.0281	88.75	9.55	Protocatechuic Acid
6	18.56	353.0867	C_16_H_18_O_9_	354.094	96.13	−3.05	5-Caffeoylquinic acid
7	18.89	197.048	C_9_H_10_O_5_	198.054	93.58	6.13	Syringic Acid
8	19.76	193.0525	C_10_H_10_O_4_	194.0597	84.19	9.47	Ferulic Acid
9	20.68	289.0721	C_15_H_14_O_6_	290.075	97.95	1.53	Catechin
10	20.81	337.0919	C_16_H_18_O_8_	338.0993	96.65	−2.49	5-O-(4-Coumaroyl)quinic acid
11	20.82	755.1986	C_33_H_40_O_20_	756.206	74.21	−7.05	Quercetin-3-O-rutinoside-rhamnoside
12	23.67	447.0899	C_21_H_20_O_11_	448.0973	85.55	−7.76	Quercetin 3-O-rhamnoside
13	24.39	609.15	C_27_H_30_O_16_	610.5	66.33	−9.06	Rutin
14	24.60	167.0364	C_8_H_8_O_4_	168.0437	89.48	8.58	Homogentisic acid
15	24.62	167.0354	C_8_H_8_O_4_	168.0437	89.48	8.58	Vanillic Acid
16	25.01	163.0415	C_9_H_8_O_3_	164.0488	89.36	8.85	*p*-Coumaric Acid
17	25.51	301.0352	C_15_H_10_O_7_	302.0424	97.84	−0.78	Morin
18	26.68	151.0416	C_8_H_8_O_3_	152.048	85.14	8.7	Vanillin
19	33.54	339.073	C_15_H_16_O_9_	340.0787	97.27	−2.22	Esculin
20	34.18	315.0511 [HCOO^−^]	C_15_H_10_O_5_	270.09	95.77	0.31	Apigenin
21	34.95	285.0408	C_15_H_10_O6	286.0482	97.91	1.73	Kaempferol

## Data Availability

The data presented in this study are available in this manuscript.

## References

[1] WFO Plant List *Ficus* L.. http://https://wfoplantlist.org/plant-list.

[2] Lansky E.P., Paavilainen H.M. (2010). Figs: The Genus Ficus.

[3] Singh D., Singh B., Goel R.K. (2011). Traditional uses, phytochemistry and pharmacology of *Ficus religiosa*: A review. J. Ethnopharmacol..

[4] Devanesan E.B., Anand A.V., Kumar P.S., Vinayagamoorthy P., Basavaraju P. (2018). Phytochemistry and Pharmacology of *Ficus religiosa*. Syst. Rev. Pharm..

[5] Sandeep, Kumar A., Sepla D., Tomer V., Gat Y., Kumar V. (2018). *Ficus religiosa*: A wholesome medicinal tree. J. Pharmacogn. Phytochem..

[6] Cheng J.-X., Zhang B.-D., Zhu W.-F., Zhang C.-F., Qin Y.-M., Abe M., Akihisa T., Liu W.-Y., Feng F., Zhang J. (2020). Traditional uses, phytochemistry, and pharmacology of *Ficus hispida* Lf: A review. J. Ethnopharmacol..

[7] Bucic-Kojic A., Planinic M., Tomas S., Jokic S., Mujic I., Bilic M., Velic D. (2011). Effect of extraction conditions on the extractability of phenolic compounds from lyophilised fig fruits (*Ficus carica* L.). Pol. J. Food Nutr. Sci..

[8] Lansky E.P., Paavilainen H.M., Pawlus A.D., Newman R.A. (2008). *Ficus* spp. (fig): Ethnobotany and potential as anticancer and anti-inflammatory agents. J. Ethnopharmacol..

[9] Salehi B., Prakash Mishra A., Nigam M., Karazhan N., Shukla I., Kiełtyka-Dadasiewicz A., Sawicka B., Głowacka A., Abu-Darwish M.S., Hussein Tarawneh A. (2021). *Ficus* plants: State of the art from a phytochemical, pharmacological, and toxicological perspective. Phytother. Res..

[10] Dell’Annunziata F., Sellitto C., Franci G., Marcotullio M.C., Piovan A., Della Marca R., Folliero V., Galdiero M., Filippelli A., Conti V. (2022). Antiviral Activity of *Ficus rubiginosa* Leaf Extracts against HSV-1, HCoV-229E and PV-1. Viruses.

[11] PlantNET (1990). Ficus Rubiginosa Desf. ex Vent.

[12] Li A.N., Li S., Zhang Y.J., Xu X.R., Chen Y.M., Li H.B. (2014). Resources and biological activities of natural polyphenols. Nutrients.

[13] Lang Y., Gao N., Zang Z., Meng X., Lin Y., Yang S., Yang Y., Jin Z., Li B. (2024). Classification and antioxidant assays of polyphenols: A review. J. Future Foods.

[14] Bharadvaja N., Gautam S., Singh H. (2023). Natural polyphenols: A promising bioactive compounds for skin care and cosmetics. Mol. Biol. Rep..

[15] Scandar S., Zadra C., Marcotullio M.C. (2023). Coriander (*Coriandrum sativum*) Polyphenols and Their Nutraceutical Value against Obesity and Metabolic Syndrome. Molecules.

[16] Zhor C., Wafaa L., Ghzaiel I., Kessas K., Zarrouk A., Ksila M., Ghrairi T., Latruffe N., Masmoudi-Kouki O., El Midaoui A. (2023). Effects of polyphenols and their metabolites on age-related diseases. Biochem. Pharmacol..

[17] Kar A., Mahar D., Biswas S., Chakraborty D., Efferth T., Panda S. (2023). Phytochemical profiling of polyphenols and thyroid stimulatory activity of *Ficus religiosa* leaf extract in 6-propyl-thiouracil-induced hypothyroid rats. J. Ethnopharmacol..

[18] Muema F.W., Kimutai F., Xu Y.-B., Zhang H., Chen G.-L., Guo M.-Q. (2021). Antioxidant and antiproliferative potentials of *Ficus glumosa* and its bioactive polyphenol metabolites Moses Mutuse Mutungi. Pharmaceuticals.

[19] Shih Y.-Z., Huang A.-J., Hou C.-Y., Jiang C.-M., Wu M.-C. (2017). The stimulating effects of polyphenol and protein fractions from jelly fig (*Ficus awkeotsang* Makino) achenes against proliferation of leukemia cells. J. Food Drug Anal..

[20] Saleh B., Hammoud R., Al-Mariri A. (2015). Antimicrobial activity of *Ficus sycomorus* L. (Moraceae) leaf and stem-bark extracts against multidrug resistant human pathogens. Herba Pol..

[21] Chhoud R., Montero F.V., Haj Romdhane M., Majdoub H., Duran Ogalla R. (2022). Phytochemical and Bioactivities of Male Flower Buds of Fruit Trees from the Southern Tunisia: Polyphenols UPLC-MS Profiles and Antioxidant Enzymatic Potential in Human Plasma of Parkinson’s Disease Patients. Chem. Afr..

[22] Hakiman M., Syed M.A., Syahida A., Maziah M. (2012). Total antioxidant, polyphenol, phenolic acid, and flavonoid content in *Ficus deltoidea* varieties. J. Med. Plants Res..

[23] Kone A.D., Mbow B., Gaye A.A., Ndoye S.F., Gaye M. (2022). *Ficus sycomorus* L. extracts: Phytochemical screening, total polyphenols and flavonoids contents, antioxidant and antibacterial activity. Sci. J. Chem..

[24] Nakilcioğlu-Taş E., Ötleş S. (2021). Influence of extraction solvents on the polyphenol contents, compositions, and antioxidant capacities of fig (*Ficus carica* L.) seeds. An. Acad. Bras. Cienc..

[25] Singh J.P., Singh B., Kaur A. (2022). Polyphenols in fig: A review on their characterisation, biochemistry during ripening, antioxidant activity and health benefits. Int. J. Food Sci. Technol..

[26] Thamburaj S., Rajagopal V., Palanivel R., Pugazhendhi S. (2022). Effect of different drying treatments on total polyphenolics content and in-vitro biological properties of *Ficus benghalensis* fruit: A comparative study. Biocatal. Agric. Biotechnol..

[27] Kittibunchakul S., Hudthagosol C., Sanporkha P., Sapwarobol S., Suttisansanee U., Sahasakul Y. (2022). Effects of Maturity and Thermal Treatment on Phenolic Profiles and In Vitro Health-Related Properties of Sacha Inchi Leaves. Plants.

[28] Korus A. (2011). Level of Vitamin C, Polyphenols, and Antioxidant and Enzymatic Activity in Three Varieties of Kale (*Brassica oleracea* L. Var. Acephala) at Different Stages of Maturity. Int. J. Food Prop..

[29] Oszmiański J., Lachowicz S., Gorzelany J., Matłok N. (2018). The effect of different maturity stages on phytochemical composition and antioxidant capacity of cranberry cultivars. Eur. Food Res. Technol..

[30] Nadeem M., Zeb A. (2018). Impact of maturity on phenolic composition and antioxidant activity of medicinally important leaves of *Ficus carica* L.. Physiol. Mol. Biol. Plants.

[31] Zolghadri S., Bahrami A., Hassan Khan M.T., Munoz-Munoz J., Garcia-Molina F., Garcia-Canovas F., Saboury A.A. (2019). A comprehensive review on tyrosinase inhibitors. J. Enzyme Inhib. Med. Chem..

[32] Hassan M., Shahzadi S., Kloczkowski A. (2023). Tyrosinase Inhibitors Naturally Present in Plants and Synthetic Modifications of These Natural Products as Anti-Melanogenic Agents: A Review. Molecules.

[33] Renda G., Barut B., Ceren R., Aydin E. (2023). In vitro tyrosinase inhibitory, DNA interaction studies, and LC? HRMS analysis of *Ficus carica* leaves. Turk. J. Chem..

[34] Rafiq M., Ilyas H., Ali A., Tarar Z., Hanif U., Javed H., Tahir T. (2021). Anti-tyrosinase and anti-oxidant potential of methanolic extracts of selected *Citrus bergamia* and *Ficus carica* parts. J. Weed Sci. Res..

[35] Suliman S., Yagi S., Elbashir A.A., Mohammed I., Hussein A., Ak G., Zengin G., Orlando G., Ferrante C. (2021). Phenolic profile, enzyme inhibition and antioxidant activities and bioinformatics analysis of leaf and stem bark of *Ficus sycomorus* L.. Process Biochem..

[36] Pucciarini L., Ianni F., Petesse V., Pellati F., Brighenti V., Volpi C., Gargaro M., Natalini B., Clementi C., Sardella R. (2019). Onion (*Allium cepa* L.) Skin: A Rich Resource of Biomolecules for the Sustainable Production of Colored Biofunctional Textiles. Molecules.

[37] Puri C., Pucciarini L., Tiecco M., Brighenti V., Volpi C., Gargaro M., Germani R., Pellati F., Sardella R., Clementi C. (2020). Use of a Zwitterionic Surfactant to Improve the Biofunctional Properties of Wool Dyed with an Onion (*Allium cepa* L.) Skin Extract. Antioxidants.

[38] Christ B., Müller K. (1960). Zur serienmäßigen Bestimmung des Gehaltes an Flavonol-Derivaten in Drogen. Arch. Pharm..

[39] Mekonnen M.M., Hoekstra A.Y. (2012). A Global Assessment of the Water Footprint of Farm Animal Products. Ecosystems.

[40] Salwinski A. Method for Determining Affinity between Ligands and a Target. Patent number WO2018055053A1 29 March 2018. https://worldwide.espacenet.com/patent/search/family/057286738/publication/EP3516395A1?q=pn%3DEP3516395A1%3F.

[41] Laosirisathian N., Saenjum C., Sirithunyalug J., Eitssayeam S., Sirithunyalug B., Chaiyana W. (2020). The Chemical Composition, Antioxidant and Anti-Tyrosinase Activities, and Irritation Properties of Sripanya *Punica granatum* Peel Extract. Cosmetics.

[42] Bhattacharya A., Sood P., Citovsky V. (2010). The roles of plant phenolics in defence and communication during *Agrobacterium* and *Rhizobium* infection. Mol. Plant Pathol..

[43] Rana A., Samtiya M., Dhewa T., Mishra V., Aluko R.E. (2022). Health benefits of polyphenols: A concise review. J. Food Biochem..

[44] Williams R.J., Spencer J.P., Rice-Evans C. (2004). Flavonoids: Antioxidants or signalling molecules?. Free Radic. Biol. Med..

[45] Piccolella S., Crescente G., Candela L., Pacifico S. (2019). Nutraceutical polyphenols: New analytical challenges and opportunities. J. Pharm. Biomed. Anal..

[46] Zhang Z., Li X., Sang S., McClements D.J., Chen L., Long J., Jiao A., Jin Z., Qiu C. (2022). Polyphenols as Plant-Based Nutraceuticals: Health Effects, Encapsulation, Nano-Delivery, and Application. Foods.

[47] Rudrapal M. (2023). Polyphenols: Food, Nutraceutical, and Nanotherapeutic Applications.

[48] André C.M., Oufir M., Hoffmann L., Hausman J.-F., Rogez H., Larondelle Y., Evers D. (2009). Influence of environment and genotype on polyphenol compounds and in vitro antioxidant capacity of native Andean potatoes (*Solanum tuberosum* L.). J. Food Compos. Anal..

[49] Shao Y., Tang F., Huang Y., Xu F., Chen Y., Tong C., Chen H., Bao J. (2014). Analysis of Genotype × Environment Interactions for Polyphenols and Antioxidant Capacity of Rice by Association Mapping. J. Agric. Food Chem..

[50] Socaci S.A., Fărcaş A.C., Diaconeasa Z.M., Vodnar D.C., Rusu B., Tofană M. (2018). Influence of the extraction solvent on phenolic content, antioxidant, antimicrobial and antimutagenic activities of brewers’ spent grain. J. Cereal Sci..

[51] Thouri A., Chahdoura H., El Arem A., Omri Hichri A., Ben Hassin R., Achour L. (2017). Effect of solvents extraction on phytochemical components and biological activities of Tunisian date seeds (var. Korkobbi and Arechti). BMC Complement. Altern. Med..

[52] Rezaei M., Ghasemi Pirbalouti A. (2019). Phytochemical, antioxidant and antibacterial properties of extracts from two spice herbs under different extraction solvents. J. Food Meas. Charact..

[53] Sulaiman S.F., Sajak A.A.B., Ooi K.L., Supriatno, Seow E.M. (2011). Effect of solvents in extracting polyphenols and antioxidants of selected raw vegetables. J. Food Compos. Anal..

[54] Boeing J.S., Barizão É.O., e Silva B.C., Montanher P.F., de Cinque Almeida V., Visentainer J.V. (2014). Evaluation of solvent effect on the extraction of phenolic compounds and antioxidant capacities from the berries: Application of principal component analysis. Chem. Cent. J..

[55] Liu Z., Bruins M.E., de Bruijn W.J.C., Vincken J.-P. (2020). A comparison of the phenolic composition of old and young tea leaves reveals a decrease in flavanols and phenolic acids and an increase in flavonols upon tea leaf maturation. J. Food Compos. Anal..

[56] Chang X., Lu Y., Lin Z., Qiu J., Guo X., Pan J., Abbasi A.M. (2018). Impact of Leaf Development Stages on Polyphenolics Profile and Antioxidant Activity in *Clausena lansium* (Lour.) Skeels. Biomed. Res. Int..

[57] Abiven S., Heim A., Schmidt M. (2011). Lignin content and chemical characteristics in maize and wheat vary between plant organs and growth stages: Consequences for assessing lignin dynamics in soil. Plant Soil.

[58] Shraim A.M., Ahmed T.A., Rahman M.M., Hijji Y.M. (2021). Determination of total flavonoid content by aluminum chloride assay: A critical evaluation. LWT.

[59] Anwar K., Rahmanto B., Triyasmono L., Rizki M., Halwany W., Lestari F. (2017). The Influence of Leaf Age on Total Phenolic, Flavonoids, and Free Radical Scavenging Capacity of *Aquilaria beccariana*. Res. J. Pharm. Biol. Chem. Sci..

[60] Sun Z., Chen D., Zhu L., Zhao Y., Lin Z., Li X., Dai W. (2022). A comprehensive study of the differences in protein expression and chemical constituents in tea leaves (*Camellia sinensis* var. sinensis) with different maturity using a combined proteomics and metabolomics method. Food Res. Int..

[61] Frankel E.N., Meyer A.S. (2000). The problems of using one-dimensional methods to evaluate multifunctional food and biological antioxidants. J. Sci. Food Agric..

[62] Apak R., Özyürek M., Güçlü K., Çapanoğlu E. (2016). Antioxidant Activity/Capacity Measurement. 2. Hydrogen Atom Transfer (HAT)-Based, Mixed-Mode (Electron Transfer (ET)/HAT), and Lipid Peroxidation Assays. J. Agric. Food Chem..

[63] Apak R., Özyürek M., Güçlü K., Çapanoğlu E. (2016). Antioxidant Activity/Capacity Measurement. 1. Classification, Physicochemical Principles, Mechanisms, and Electron Transfer (ET)-Based Assays. J. Agric. Food Chem..

[64] Zhang X., Lv H., Li Z., Jiang K., Lee M.R. (2015). HPLC/QTOF-MS/MS application to investigate phenolic constituents from *Ficus pandurata* H. aerial roots. Biomed. Chromatogr..

[65] Kıvrak Ş., Kıvrak İ., Karababa E. (2018). Analytical evaluation of phenolic compounds and minerals of *Opuntia robusta* J.C. Wendl. and *Opuntia ficus-barbarica* A. Berger. Int. J. Food Prop..

[66] Si Y.-X., Yin S.-J., Oh S., Wang Z.-J., Ye S., Yan L., Yang J.-M., Park Y.-D., Lee J., Qian G.-Y. (2012). An Integrated Study of Tyrosinase Inhibition by Rutin: Progress using a Computational Simulation. J. Biomol. Struct. Dyn..

[67] El-Nashar H.A.S., El-Din M.I.G., Hritcu L., Eldahshan O.A. (2021). Insights on the Inhibitory Power of Flavonoids on Tyrosinase Activity: A Survey from 2016 to 2021. Molecules.

[68] Truong X.T., Park S.-H., Lee Y.-G., Jeong H.Y., Moon J.-H., Jeon T.-I. (2017). Protocatechuic Acid from Pear Inhibits Melanogenesis in Melanoma Cells. Int. J. Mol. Sci..

